# Cell‐free DNA screening for rare autosomal trisomies and segmental chromosome imbalances

**DOI:** 10.1002/pd.6233

**Published:** 2022-09-22

**Authors:** Yvette C. Raymond, Shavi Fernando, Melody Menezes, Simon Meagher, Ben W. Mol, Andrew McLennan, Fergus Scott, Karen Mizia, Karen Carey, Gabrielle Fleming, Daniel Lorber Rolnik

**Affiliations:** ^1^ Department of Obstetrics and Gynecology Monash University Clayton Victoria Australia; ^2^ Monash Women's Monash Health Clayton Victoria Australia; ^3^ Monash Ultrasound for Women Melbourne Victoria Australia; ^4^ Department of Pediatrics The University of Melbourne Melbourne Victoria Australia; ^5^ Aberdeen Centre for Women's Health Research University of Aberdeen Aberdeen UK; ^6^ Sydney Ultrasound for Women Sydney New South Wales Australia; ^7^ Discipline of Obstetrics, Gynaecology and Neonatology The University of Sydney Sydney New South Wales Australia; ^8^ School of Women's and Children's Health University of New South Wales Sydney New South Wales Australia; ^9^ Ultrasound Care Sydney New South Wales Australia; ^10^ Australian National University Canberra Australia

## Abstract

**Objective:**

To assess the outcomes of pregnancies at high‐risk for rare autosomal trisomies (RATs) and segmental imbalances (SIs) on cell‐free DNA (cfDNA) screening.

**Method:**

A retrospective study of women who underwent cfDNA screening between September 2019 and July 2021 at three ultrasound services in Australia. Positive predictive values (PPVs) were calculated using fetal chromosomal analysis.

**Results:**

Among 23,857 women screened, there were 93 high‐risk results for RATs (0.39%) and 82 for SIs (0.34%). The PPVs were 3.8% (3/78, 95% CI 0.8%–10.8%) for RATs and 19.1% (13/68, 95% CI 10.6%–30.5%) for SIs. If fetuses with structural anomalies were also counted as true‐positive cases, the PPV for RATS increased to 8.5% (7/82, 95% CI 3.5%–16.8%). Among 85 discordant cases with birth outcomes available (65.4%), discordant positive RATs had a significantly higher proportion of infants born below the 10th and 3rd birthweight percentiles than expected (19.6% (*p* = 0.022) and 9.8% (*p* = 0.004), respectively), which was not observed in the SI group (2.9% < 10th (*p* = 0.168) and 0.0% <3rd (*p* = 0.305)).

**Conclusion:**

The PPVs for SI and RAT results are low, except when a structural abnormality is also present. Discordant positive RATs are associated with growth restriction.

## Key points

What is already known about this topic?

- Prenatal cell‐free DNA (cfDNA) screening has high accuracy in screening for trisomies 21, 18 and 13.
- The positive predictive values of cfDNA screening for rare autosomal. Trisomies rare autosomal trisomies (RATs) and segmental imbalances (SIs) are lower than that for common autosomal trisomies.
- Fetal exclusion of chromosomal anomaly following high‐risk cfDNA screen may indicate confined placental involvement.

What does this study add?

- True‐positive (fetal) results are infrequent in women screened high‐risk for SIs and RATs in the absence of ultrasound findings.
- Discordant positive cfDNA results for RAT may indicate an increased risk of growth restriction. There does not appear to be any risk of growth restriction after a discordant positive SI result.

## 1 INTRODUCTION

Cell‐free DNA (cfDNA) screening, commonly referred to as non‐invasive prenatal testing, is a method of prenatal aneuploidy screening. CfDNA released by cytotrophoblast and nucleated maternal cells is extracted from a maternal plasma sample after 10 weeks' gestation, and bioinformatic analysis of the sequences can indicate chromosomal anomalies.^1^ Importantly, the screening performance of cfDNA for fetal aneuploidy relies on concordance between the fetal and placental genomes.^2^, ^3^ Compared to alternatives, cfDNA screening has demonstrated impressive detection rates and accuracy in predicting trisomies 21, 18 and 13. The sensitivity of cfDNA for trisomy 21, 18 and 13 is 99.7%, 97.9% and 99.0% respectively, with a false‐positive rate of 0.04% each.^4^ The PPVs for trisomy 21, 18 and 13 are 92.4%, 84.6% and 43.9%.^5^

More recently, cfDNA screening has been expanded to report anomalies for all chromosomes, as opposed to targeted panels which assess only chromosomes 21, 18, 13 and the sex chromosomes. This expanded test additionally screens for rare autosomal trisomies (RATs) and segmental imbalances (SIs). Rare autosomal trisomies may result from either mitotic (most common for chromosomes 2, 3, 7, 8), or meiotic errors (commonly chromosomes 14, 15, 16, 22).^5^, ^6^, ^7^ Non‐mosaic RATs are a rare finding in fetal tissues beyond early pregnancy, as most of these anomalies are life‐limiting and miscarry early.^6^, ^8^, ^9^, ^10^ Conversely, many SIs do not result in miscarriage, with chromosomal deletions (excluding microdeletions) estimated to be present in 0.019% of live births, and duplications in 0.007%.^11^ The clinical implications for many of these anomalies are poorly mapped, as phenotypic manifestations are highly variable and depend on the gene region involved as well as the size of the aberration, but range from benign to profoundly disabling.^12^, ^13^, ^14^

As the use of whole‐genome prenatal screening increases globally, attention has focused on the accuracy in identifying these rare anomalies, given that predictive value of screening is related to disease prevalence. Given their rarity in fetal tissues, it is theorized that the majority of RATs identified by cfDNA are confined to the placenta, in full or mosaic form.^15^, ^16^ This is a biologically plausible explanation for higher rates of adverse pregnancy outcomes, including fetal growth restriction (FGR), amongst women who screen high‐risk for RATs independent of fetal karyotype, although there is debate regarding the strength of this association.^6^, ^17^, ^18^, ^19^, ^20^, ^21^, ^22^ Evidence for the performance of cfDNA for fetal SIs is also limited.^18^, ^23^, ^24^, ^25^

The aims of this study are to assess the predictive accuracy of cfDNA in screening for RATs and SIs, and to investigate the association between discordant positive results and placental insufficiency.

## 2 MATERIAL AND METHODS

### 2.1 Study design and governance

This was a retrospective study performed at three fetal medicine practices in Australia (two in Sydney and one in Melbourne). Women underwent cfDNA screening as part of routine clinical care governed by their respective obstetric care providers, as either a primary screening method or subsequent to a high‐risk result from other methods of screening or ultrasound findings. Screening was patient‐funded. Ethical approval for this study was obtained from the Monash University Human Research Ethics Committee (project ID 26175).

### 2.2 Inclusion and exclusion criteria

We studied women who received a high‐risk cfDNA result for either a RAT or SI between September 2019 and July 2021. Women aged over 18 years with high‐risk cfDNA results and available post‐screening outcomes were included. Women aged under 18 years were excluded, due to capacity to consent, as were those women who opted for screening of common aneuploidies only, rather than genome‐wide cfDNA screening. Women with a multiple pregnancy were not excluded.

Some participants between November 2019 and December 2020 have been included in another publication from our group,^26^ however such publication focused on the impact of fibroids on test accuracy rather than on overall test accuracy and pregnancy outcomes, and were therefore not considered duplicated data.

### 2.3 Procedures

Maternal plasma samples were collected at or after 10 weeks' gestation. All providers offered screening with next‐generation massively parallel sequencing technology using the Illumina VeriSeq v2 platform (Illumina Inc.) to detect genetic aberrations larger than 7Mb on autosomal chromosomes. Microdeletion/duplication syndromes are not included in these screening panels.

Data were collected through audit of routinely kept records at each participating site. Recorded variables included maternal age, indication for cfDNA screening, and pregnancy biomarker results including pregnancy‐associated plasma protein A (PAPP‐A) recorded from other prenatal screening investigations at the participating fetal medicine practices, where available. Genetic confirmation was sought for patients who accepted diagnostic investigations, and infant phenotype or pregnancy outcomes for those who declined. Infant phenotype was also investigated for true‐positive cases. Birth outcomes were obtained for discordant positive cases to evaluate the ramifications of putative confined placental mosaicism (CPM). Follow‐up involved a combination of medical record auditing and participant contact via telephone interview or an online survey hosted by RedCap (v10.6.21, 2021, Vanderbilt University).

### 2.4 Statistical analysis

Categorical variables are expressed as numbers and proportions and continuous variables in means and standard deviations (SD) or medians and interquartile ranges (IQR) depending on the frequency distribution. Differences in means and medians were analysed using independent‐samples *t*‐tests and Wilcoxon rank‐sum test, as appropriate. The difference in proportions of pregnancies featuring ultrasound anomalies depending on screening concordance was assessed with the Chi‐square test.

Positive predictive value was calculated with exact binomial 95% confidence intervals (CI). Calculations were based on fetal genetic confirmation, thus unconfirmed karyotypes, where only phenotypes were available, were not included in the primary analysis. Women in whom diagnostic testing revealed uniparental disomy (UPD) were classified as ‘true‐positive’ RATs, as trisomy was once most likely present in the embryo prior to trisomic rescue.^26^ Accordingly, results which were confirmed to be attributable to CPM (mosaic trisomy detected on chorionic villus sampling (CVS) followed by normal amniocentesis) were considered ‘discordant positive’, as the analysis pertains to true fetal anomalies. For SIs, screening results were considered concordant to diagnostic investigations providing the aberration was located on the same chromosome. Where women received high‐risk results for multiple SIs, variants located on different chromosomes were considered individually.

Birthweight percentiles were calculated using the Australian national birthweight by sex and gestational age chart by Dobbins et al.^27^ The Wilcoxon signed‐rank test was used to assess for differences between the distribution of birthweight percentiles in each anomaly group and the expected birthweight percentile distribution in the general population, centered at the 50th percentile. For RATs, the median birthweight percentiles were compared between the chromosome trisomies most likely of mitotic origin (2, 3, 7, 8), versus meiotic (14, 15, 16, 22), with grouping guided by the current literature.^5^, ^6^, ^7^ Statistical analyses were not conducted for this comparison, however, as we do not have confirmation of trisomy origin. Proportions of neonates with birthweight below the 10th and the third percentiles were compared with the expected proportions in the general population (10% and 3%, respectively) using one‐sample tests of proportions.

Two‐sided *p*‐values below 0.05 were considered statistically significant. Statistical analyses were conducted in Stata (StataCorp. 2021. Stata Statistical Software: Release 17. College Station).

## 3 RESULTS

**FIGURE 1:**
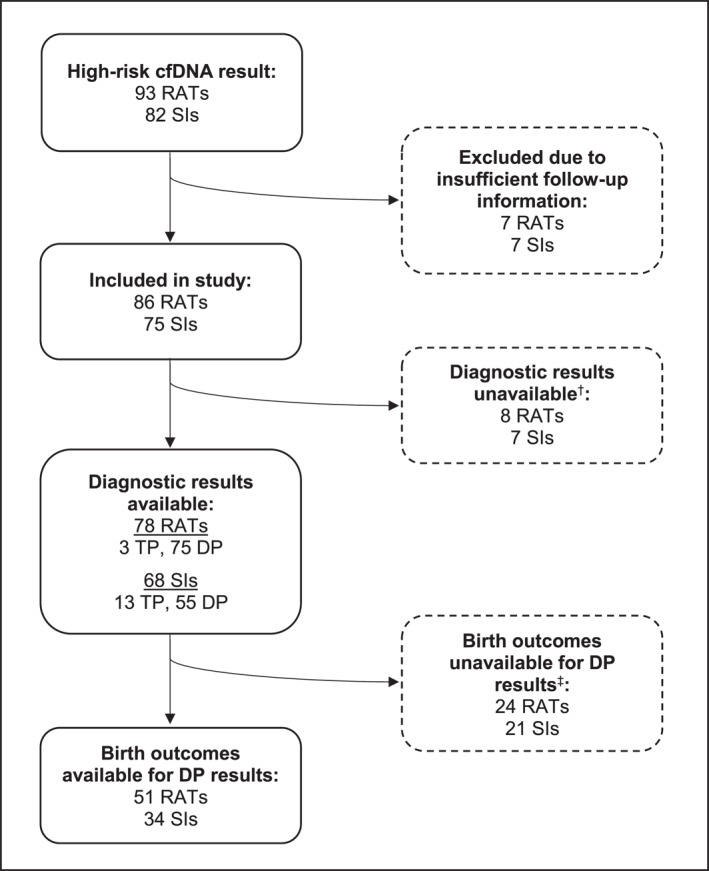
Flow chart of data collection. †Parents declined either prenatal or postnatal diagnostic testing. Four RAT cases had structural/phenotype or chorionic villus sampling anomalies. ‡Participants delivered at inaccessible sites or still pregnant at the time of data collection. cfDNA, cell‐free DNA; DP, discordant positive; RAT, rare autosomal trisomy; SI, segmental imbalance; TP, true‐positive

Among 23,857 genome‐wide cfDNA screens conducted across all sites, there were 93 high‐risk results for RATs and 82 high‐risk results for SIs (screen‐positive rates of 0.39% (95% CI 0.31%–0.48%) and 0.34% (95% CI 0.27%–0.43%), respectively) of these, seven RATs (7.5%) and seven SIs (8.3%) results were excluded due to failure to obtain any post‐screening information, leaving 161 high‐risk results in the study (86 RATs and 75 SIs, Figure 1).

Maternal characteristics are summarized in Table 1. The mean maternal age of participants was 34.8 years (95% CI 34.3–35.4). Women aged over 35 years accounted for 46.6% of the screen‐positive population (45.3% of RATs and 48.0% of SIs). Maternal age did not differ significantly between the high‐risk RAT and SI groups (*p* = 0.879). Three women (1.9%) underwent cfDNA screening after a high‐risk first trimester combined screening result, all with high‐risk results for RATs, while the remaining 158 women elected cfDNA as a primary screening method (98.1%).

**TABLE 1:** Maternal and pregnancy characteristics of the study participants

	RATs *n* = 86	SIs *n* = 75
Age in years, mean (SD)*	34.8 (3.5)	34.8 (3.7)
BMI in kg/m^2^, median (IQR)	22.5 (21.2–26.2)	23.9 (21.7–27.9)
Conception method, *n* (%)		
Spontaneous	76 (88.4)	66 (88.0)
IVF	10 (11.6)	9 (12.0)
Multiple pregnancy, *n* (%)		
Singleton	84 (97.7)	75 (100.0)
Twin	0 (0.0)	0 (0.0)
Triplet	2 (2.3)	0 (0.0)
Gestation at screening in weeks, median (IQR)	11.1 (10.7–11.6)	11 (10.6–11.6)
Diagnostic testing choice, *n* (%)		
Chorionic villous sampling	4^a^ (4.7)	7 (9.3)
Amniocentesis	72 (83.7)	55 (73.4)
Postnatal testing	3 (3.5)	5 (6.7)
Combination	1^b^ (1.2)	1^c^ (1.3)
Declined	6 (6.9)	7 (9.3)

Abbreviations: BMI, body mass index; IQR, interquartile range; IVF, in vitro fertilization; RAT, rare autosomal trisomy; SI, segmental imbalances; SD, standard deviation.

^a^ Results of chorionic villus sampling were concordant with screening results in two instances, however these were not considered in diagnostic totals for positive predictive value analyses given the lack of fetal genome confirmation and the high likelihood of confined placental mosaicism.

^b^ Chorionic villus sampling and postnatal testing.

^c^ Chorionic villus sampling and amniocentesis.

*p‐value for the difference = 0.879.

Fetal genome information from diagnostic testing was available for 146 results (90.7%), with only pregnancy outcomes or infant phenotype available for the remaining 9.3%. In two cases involving a high‐risk RAT result, CVS was undertaken and revealed placental mosaicism, but amniocentesis and postnatal testing were declined, thus these women were considered ‘unconfirmed’ due to the considerable likelihood of CPM.

**FIGURE 2:**
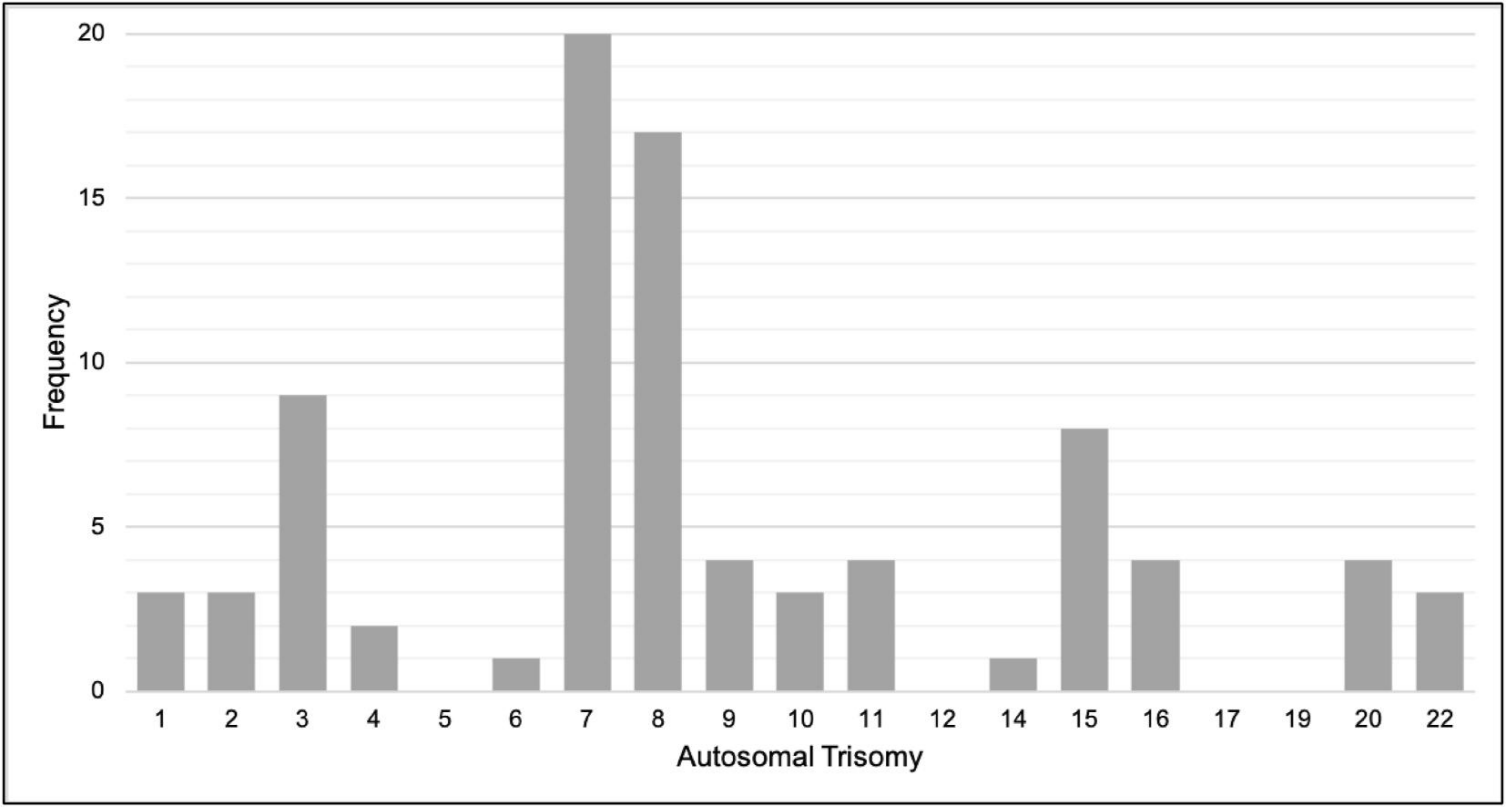
Number of rare autosomal trisomies (RATs) detected, by chromosome

Trisomy 7 was the most frequently reported RAT (*n* = 20, 23.3%) (Figure 2) of the 78 women with a high‐risk RAT with diagnostic confirmation available, three were true‐positive results (one trisomy 16, two UPD cases involving chromosomes 6 and 16), yielding a PPV of 3.8% (95% CI 0.8%–10.8%) and an incidence of 1 in 7952 (0.013%, assuming that there were no false‐negative results). Results of UPD studies were recorded for 26 high‐risk RAT results, most frequently for trisomy 7 (*n* = 13) and 15 (*n* = 7). Two of the three true‐positive RAT results resulted in a live‐birth. Both involved UPD (UPD6 and UPD16). Delivery was expedited in both women before 30 weeks' gestation, due to FGR with oligohydramnios in the woman with UPD6, and for FGR and severe pre‐eclampsia in the woman with UPD16. In addition, the UPD16 affected infant had a cleft palate and unilateral ocular coloboma. At the time follow‐up was conducted both infants were less than 6 months old and were affected by multiple complications of prematurity. The remaining woman with a true‐positive RAT had amniocentesis confirmation of mosaic fetal trisomy 16 and subsequently terminated at 17 weeks.

Segmental deletions accounted for 52.0% of SIs (*n* = 39/75), and segmental duplications for 48.0% (*n* = 36/75). Among the high‐risk results, segmental deletions ranged in size between 8 and 78Mb (median 21.6 Mb), and segmental duplications between 8 and 136.3 Mb (median 22.1 Mb). 13 of the 68 high‐risk results for SIs with diagnostic outcomes were true‐positive cases, yielding a PPV of 19.1% (95% CI 10.6%–30.5%) and an incidence of 1 in 1835 (0.055%). Complete information regarding all SIs can be found in Supplementary Materials: Table S1. There were no true‐positive SI results which resulted in a live birth, as all 13 women opted for termination.

Four women received results suggesting two or more aberrations on different chromosomes (three women with two aberrations, one woman with three aberrations), although only one of these women had an affected fetus, with one of the two detected duplications later revealed to be paternally inherited. Another woman received a cfDNA result suggesting two duplications in separate regions of chromosome 1, which was discordant with diagnostic testing.

The presence of fetal anomalies on ultrasound was associated with 19 times higher odds of true‐positive RAT or SI results (odds ratio 19.6, 95% CI 5.8–66.4, *p* < 0.001) compared to women with no abnormal sonographic features. When restricted to women with significant ultrasound anomalies, the PPV increased to 28.6% for RATs (2/7, 95% CI 3.7%–71.0%) and 70.0% for SIs (7/10, 95% CI 34.8%–93.3%). One of the three true‐positive RAT results (mosaic trisomy 16) was not accompanied by ultrasound anomalies, although ultrasound information for this pregnancy was only available up to 15 weeks' gestation. Six of 13 (46.2%) true‐positive SI results had unremarkable ultrasound findings, with 20 week morphology ultrasound results available for all but two of these pregnancies.

Of the 15 women without fetal genetic confirmation, six elected termination of pregnancy (five RATs, one SI). Three of these RAT results were informed by either ultrasound anomalies or abnormal CVS results, while three women elected termination of pregnancy immediately following their high‐risk cfDNA result, despite receiving genetic counselling. The remaining nine unconfirmed pregnancies resulted in live births. One child who had screened high‐risk for trisomy 7 developed epileptic seizures before 12 months of age. The remaining infants were phenotypically normal. Assuming fetal ultrasound anomalies or significant infant phenotype represents a true‐positive cfDNA result in the absence of genetic confirmation, the PPV for RATs would be 8.5% (7/82, 95% CI 3.5%–16.8%), with an incidence of 1 in 3408 (0.029%), and unchanged for SIs.

Birth outcomes were obtained for 65.4% discordant positive results (68.0% of RATs, 61.8% of SIs) (Figure 1). The available outcomes are summarized in Table 2. Notably, there was one instance of septic miscarriage of a fetus with a normal karyotype following CVS, indicated by a high‐risk cfDNA result for a SI. The distributions of birthweight percentiles for discordant positive SIs and discordant positive RATs were not significantly different from that expected in the general population. However, there were significantly higher proportions of birthweights below the 10th and 3rd percentiles in the discordant positive RAT group (<10th percentile: 2 × T2, 3 × T7, 1 × T8, 1 × T9, 2 × T15, 1 × T22, <3rd percentile: 2 × T2, 1 × T7, 1 ×  × T15, 1 × T22), but not in the discordant positive SI group. The median birthweight percentile was lower amongst RATs most frequently of meiotic (median 24th percentile, IQR 1st to 56th percentile), compared to mitotic (median 41st percentile, IQR 22nd to 66th percentile), origin.

**TABLE 2:** Birth outcomes of pregnancies with discordant positive cfDNA results

	RATs	SIs	General obstetric population
	*n* = 51	*n* = 34	
Gestation at birth in weeks, median (IQR)	39.0 (38.1–39.3)	38.4 (37.9–39.0)	‐
Birthweight in grams, median (IQR)	3180 (2870–3500)	3240 (3068–3675)	‐
Birthweight percentile, median (IQR)	43.9 (17.3–61.1)	47.8 (26.6–66.6)	50.0 (25.0–75.0)
*p‐value	0.135	0.839	
Proportion with birthweight <10th percentile, *n* (%)	10 (19.6)	1 (2.9)	− (10.0)
*p‐value	0.022	0.168	
Proportion with birthweight <3rd percentile, *n* (%)	5 (9.8)	0 (0.0)	− (3.0)
*p‐value	0.004	0.305	

Abbreviations: cfDNA, cell‐free DNA; RAT, rare autosomal trisomy; SI, segmental imbalance; IQR, interquartile range.

*p‐value for difference from General Obstetric Population.

PAPP‐A values were available for 72.2% of the study participants (*n* = 117/162). The median value was significantly lower in discordant positive RAT pregnancies affected by FGR (0.40 multiples of the median (MoM), IQR 0.11 to 0.64 MoM) than in discordant positive RATs with normal birthweights (0.84 MoM, IQR 0.64 to 1.25 MoM) (*p* = 0.020).

Outside of the RAT and SI cohorts, monosomy 2 and monosomy 14 were also observed. The fetus screened high‐risk for monosomy 14 was found to have no abnormalities on amniocentesis and resulted in a healthy neonate, while the woman with a high‐risk monosomy 2 result declined diagnostic testing in favor of immediate termination of pregnancy.

## 4 DISCUSSION

### 4.1 Main findings and interpretation

In this large multicenter study, there were confirmed fetal genetic anomalies in almost 4% of women at high‐risk for a RAT on cfDNA screening where complete diagnostic information was available. This rose to 8.5% when also considering pregnancies with incomplete genetic information where fetal structural or infant anomalies were identified, most of which terminated without diagnostic investigations. The PPV of a high‐risk result for a SI was 19.1%. The odds of fetal confirmation of a high‐risk RAT or SI cfDNA result were 19.6 times greater in the presence of ultrasound anomalies than those without.^28^

The low PPV observed in the RAT group can be attributed to our specification of true‐positive status only applying to fetal, not placental, confirmation of aneuploidy. Fetal confirmation of a non‐mosaic RAT beyond 10 weeks' gestation is exceedingly rare as these anomalies typically cause early miscarriage.^6^, ^8^ Therefore, when a RAT is detected by cfDNA screening it is probable that the aneuploidy is confined to the placenta, or less commonly, that the fetus is mosaic.^15^, ^16^ The lower PPV of cfDNA screening for SIs observed in this study compared to common aneuploidies may also be attributable to confined placental involvement, in addition to test inaccuracies arising from sporadic technical issues.

Women who received a discordant positive result for a RAT, which may indicate CPM, had a higher proportion of small neonates than expected in the general population. The possibility of CPM may help explain this finding, if this placental aneuploidy results in placental dysfunction. While not formally analyzed as we do not have confirmation of aneuploidy origin, the median birthweight percentile was considerably lower in the likely meiotic, compared to likely mitotic, subgroup. Meiotic placental aneuploidy most commonly affects both the cytotrophoblast and the mesenchymal core (type 3 CPM), and has known adverse effects on fetal growth.^29^

### 4.2 Comparison with previous studies

The PPV of cfDNA screening for RATs in this study concords with the existing literature. A large study from Belgium investigating cfDNA screening conducted by Van Den Bogaert et al. reported a PPV of 4.1% for mosaic fetal trisomy which increased to 5.3% when including UPD as true‐positives, while preliminary results from the ongoing TRIDENT‐2 study revealed a value of 6.2%.^5^, ^30^

The literature regarding the PPV of cfDNA screening for SIs is more variable than for RATs. The study by Van Den Bogaert et al., and the TRIDENT‐2 study yielded higher values than observed in this study, of 46.7% and 31.9%, respectively.^5^, ^30^ This may be partially attributable to a lower diagnostic follow‐up rate in Van Den Ben Bogaert et al., but is likely resultant from discrepancies across studies between the baseline risk of populations screened and the technical screening methods used. Chance is also a possible cause of this observed discrepancy between these results, given the rarity of SIs and the scarcity of high‐risk results across even large study populations.

The mean maternal age of women in our high‐risk cohort was higher than the general Australian obstetric population (34.8 vs 29.4 years), but similar to that of the overall population undergoing cfDNA screening observed in our previous study during a similar time window.^26^, ^31^ The mean maternal age was similar between the RAT and SI groups. This is explainable by the predominance of RATs most frequently arising from mitotic errors in our study, which like SIs and unlike meiotic errors, do not seem to be related to maternal age.^32^

### 4.3 Clinical implications

At best, approximately 1 in 13 pregnancies that screen positive for RAT will actually have fetal aneuploidy. The rate of fetal confirmation increases in the presence of ultrasound anomalies, although fetal anomalies detected before cfDNA screening may be more appropriately investigated by a diagnostic procedure rather than by cfDNA screening.^33^

The association between high‐risk RAT results and FGR warrants more rigorous growth monitoring in these pregnancies. A discordant positive RAT result in combination with low PAPP‐A levels indicates greater risk of FGR than with normal PAPP‐A, although a relationship between low PAPP‐A and FGR exists independent of cfDNA screening results.^34^ Another potential indicator of placental insufficiency is the origin of aneuploidy (mitotic or meiotic) suggested by the implicated chromosome, but this is not routinely considered in current cfDNA screening.^35^, ^36^

The clinical utility of cfDNA screening for SIs is complex. Although suggested improvements for the provision of genome‐wide cfDNA screening include restricting referrals for invasive diagnostic testing to high‐risk results accompanied by ultrasound anomalies due to the lower PPV for rare anomalies, this approach risks misclassifying clinically significant true‐positive results as many SIs may not present with abnormal ultrasound findings, as demonstrated in the current study.^37^ However, even when successfully identified, the variable phenotypic consequences are a challenge for genetic counselling.^38^, ^39^

Another important consideration is that the frequency of inaccurate cfDNA results increases with every condition added to the screening panel. Higher screen‐positive rates will likely lead more women to be referred for invasive diagnostic testing with its low but non‐negligible procedure‐related risks, as demonstrated by the woman with septic miscarriage in this study.^40^ Van Den Bogaert et al., noted that while rates of invasive testing have declined alongside the increasing uptake of first‐line cfDNA screening, they still exceed what would be necessary to capture all pregnancies with fetal aneuploidy in the population, which authors partially attribute to the reporting of genome‐wide findings.^5^

Parental anxiety following a high‐risk cfDNA result is also not a harm which should be overlooked and may persist even after normal diagnostic results.^41^, ^42^ This is evidenced by the three pregnancy terminations in this study which were guided by cfDNA alone due to parental anxiety, mostly in concert with previous adverse pregnancy outcomes, despite the low PPV of cfDNA screening for rare abnormalities. This finding is of concern as all participants in this study had access to specialist fetal medicine and genetic counselling, thus the frequency of pregnancy termination indicated solely by abnormal cfDNA results is potentially greater under non‐specialist care than that captured in this study. As a result of concerns regarding the clinical implementation of genome‐wide screening, the U.S Food and Drug Administration issued a safety communication in April, 2022 expressing caution over the interpretation of these results.^43^

### 4.4 Limitations

The main limitations of this study are attributable to incomplete or insufficient pregnancy and infant outcome information, which introduces risk of ascertainment bias. Without complete diagnostic confirmation for the entire high‐risk group, it is possible that our PPVs are biased by confounding clinical factors which influence a woman's decision to pursue additional investigations and diagnostic testing. Similarly, our findings pertaining to birth outcomes may have been affected by ascertainment bias due to incomplete follow‐up of the entire population, as pregnancies screened high‐risk for anomalies with evidenced adverse outcomes, such as trisomy 16, would likely demand closer clinical monitoring.

Calculation of test sensitivity and specificity would have only been possible with complete genetic follow‐up of women with high and low‐risk results, which is often not feasible in clinical practice. Therefore, we limited the analysis to the predictive value of a positive test, which is arguably the most important accuracy measure from an individual patient's perspective.^44^

Another limitation of this study arises when drawing conclusions regarding the risk of FGR associated with discordant positive cfDNA results. While we hypothesize that this risk is attributable to placental aneuploidy, particularly in the presence of meiotic RATs, it is impossible to ascertain whether discordant positive results actually arise from the placenta or are sporadic inaccuracies without conducting detailed placental cytogenetic analyses, which we were unable to undertake in this study.

## 5 CONCLUSION

Decisions regarding screening with expanded cfDNA panels at both the population and individual patient level should take into account the harms of increased discordant positive results along with the benefits of detection of significant rare chromosomal abnormalities that would otherwise be missed prenatally. Closer third trimester monitoring is indicated for high‐risk RAT results even following exclusion of fetal aneuploidy due to the risk of placental insufficiency.

## CONFLICT OF INTEREST

Ben W. Mol is supported by a NHMRC Investigator grant (GNT1176437). Ben W. Mol reports consultancy for ObsEva and Merck and travel support and research grants from Merck. The authors declare no conflict of interest.

## Supporting information

Table S1

## Data Availability

The data gathered in this study has not been made publicly available to protect the privacy of participants. For data enquiries, please contact corresponding author Yvette Raymond.
