# Supplementary material for: Cell‐free DNA screening for rare autosomal trisomies and segmental chromosome imbalances
Source: Prenat Diagn. 2022 Sep 22;42(11):1349–57. doi: 10.1002/pd.6233 (PMC9826090; doi:10.1002/pd.6233)
Supplement: Supplementary file 1 — Table S1 [file PD-42-1349-s001.pdf]

Supplementary Table 1. Details of cases of high-risk cfDNA results for segmental chromosomal imbalances.

Case	Type	Chromosome	Region	Size (Mb)	Diagnostic testing	Diagnostic Result	Classification	Parental Testing	Ultrasound anomalies	Outcome
1	del	1	p(32.2-31.3)	9.6	Amniocentesis	11.2Mb del in chromosome 1p(32.2-31.2)	True-positive	NAD		TOP
2	del	1	q	17.4	Declined		Unconfirmed			NLB
3	del	1	terminal deletion		Declined		Unconfirmed		Uterine fibroids	NLB
4	del	2	q(21.2-22.3)	13.5	Amniocentesis	NAD	Discordant-positive			Unconfirmed
5	del	2	p(25.3-24.1)	20.7	Amniocentesis	NAD	Discordant-positive	NAD		NLB
6	del	2	q(37.1-37.3)	9	Amniocentesis	NAD	Discordant-positive	Maternal mosaic del 2q(37.1-37.3) (16%)		NLB
7	del	3	p(26.3-21.2)	50.8	Amniocentesis	NAD	Discordant-positive			NLB
8	del	3	q(24 -26.33)	31.4	Amniocentesis	NAD	Discordant-positive	NAD	Uterine fibroid	NLB
9	del	4	q(31.21-35.2)	78	Amniocentesis	78Mb del 4q(13.3-31.3), and 38Mb del 4(q31.3-35.2)	True-positive			TOP
10	del	4	q(12-13.3)	15.2	Amniocentesis	del 4q(12-13.3)	True-positive	NAD		TOP
11	del	5	q(14.3-31.1)	48.5	Amniocentesis	NAD	Discordant-positive	NAD		Unconfirmed
12	del	5	p13.1-q11.1	10.7	Amniocentesis	NAD	Discordant-positive		Retroplacental haemorrhage, uterine fibroids	NLB
13	del	6	q(12-14.1)	12.9	Amniocentesis	del 6q(12-14.1)	True-positive	NAD		TOP
14	del	6	q(24.1-25.2)	13.7	CVS	Del 6p24.1-q25.2	True-positive		Raised NT and subcutaneous oedema	TOP
15	del	7	q(22.1-33)	34.2	Amniocentesis	NAD	Discordant-positive	NAD	Uterine fibroids	NLB
16	del	7	q(21.3-34)	47.9	Amniocentesis	NAD	Discordant-positive		Uterine fibroid	NLB
17	del	7	q(22.3-31.2)	10.7	CVS	NAD	Discordant-positive		Uterine fibroid	Septic miscarriage post CVS
18	del	7	q(21.12-32.1)	41.2	Declined		Unconfirmed	NAD	Uterine fibroids	NLB
19	del	7	p11.2-q31.2	57.6	CVS	NAD	Discordant-positive		Uterine fibroids	NLB
20	del	8	q(12.1-13.3)	13.3	Postnatal	Del in chr8	True-positive	NAD	Hydropic fetus, subcutaneous edema with large cystic hygroma, cardiac and intracranial malformation	TOP
21	del	8	q(23.3-24.13)	8.6	Amniocentesis	Del 8q(23.3-24.13)	True-positive			TOP
22	del	8	p12-q11.23	18.4	Amniocentesis	NAD	Discordant-positive	NAD		Unconfirmed
23	del	8	p(23.3-12)		Amniocentesis	NAD	Discordant-positive			NLB
24	del	9	p(22.3-21.1)	15.1	Postnatal	NAD	Discordant-positive		Single umbilical artery, right talipes	Live birth, neonate <10th birthweight percentile

Supplementary Table 1. Details of cases of high-risk cfDNA results for segmental chromosomal imbalances.

Case	Type	Chromosome	Region	Size (Mb)	Diagnostic testing	Diagnostic Result	Classification	Parental Testing	Ultrasound anomalies	Outcome
25†	del	9	p(24.3-21.2)	26	CVS	16.9Mb Del 9p(24.3-22.2), 34.1Mb Dup 12p(13.33-11.1)	True-positive	Paternal t(9;12)(p22;p11.1)	Omphalocele, unusual facial profile	TOP
26	del	10	q(25.2-26.3)	21.6	Amniocentesis	NAD	Discordant-positive	NAD		NLB
27	del	10	q	23.7	Amniocentesis	NAD	Discordant-positive			NLB
28	del	10	q(25.1-26.3)	24.1	Amniocentesis	NAD	Discordant-positive	NAD		NLB
29	del	10	q(25.2-26.3)	22.7	Amniocentesis	NAD	Discordant-positive	NAD		NLB
30	del	10	q(22.1-26.2)	56	Postnatal	219kb del 4q31.3	Discordant-positive	Maternal 219Kb Del 4q31.3	Uterine fibroids	NLB
31	del	10	q(25.2-26.3)	22.2	Amniocentesis	NAD	Discordant-positive	NAD		NLB
32	del	12	q(21.2-24.33)	54.4	Product of Conception	418kb Dup 5q11.2, no changes on Chr12	Discordant-positive		Fetal biometry all <1st centile. Normal anatomy	Miscarriage at 15/40
33	del	12	q(21.1-23.3)	35.2	Amniocentesis	NAD	Discordant-positive	NAD	Uterine fibroid	NLB
34†	del	14	q(24.2-32.33)	36.2	Amniocentesis	NAD	Discordant-positive	NAD	Uterine fibroid	Unconfirmed
34†	del	16	q(23.1-23.3)	8	Amniocentesis	NAD	Discordant-positive	NAD	Uterine fibroid	Unconfirmed
35	del	14	q(21.2-24.1)	20.1	Amniocentesis	NAD	Discordant-positive		Uterine fibroids	NLB
36†	del	18	p(11.32-11.21)	11	Amniocentesis	NAD	Discordant-positive			NLB
37	del	18	q	59.1	Product of Conception	NAD	Discordant-positive			TOP due to maternal anxiety following significant bleed at 12/40
38	del	20	q(11.21-13.13)	18.4	Amniocentesis	NAD	Discordant-positive	NAD	Borderline NT	NLB
25†	dup	12	p13.33-q12	40	CVS	16.9Mb Del 9p(24.3-22.2), 34.1Mb Dup 12p(13.33-11.1)	True-positive	Paternal t(9;12)(p22;p11.1)	Omphalocele, abnormal facial profile	TOP
34†	dup	12	q(15-24.33)	62.2	Amniocentesis	NAD	Discordant-positive	NAD	Uterine fibroid	Unconfirmed
36†	dup	20	p(13-11.1)	25.9	Amniocentesis	NAD	Discordant-positive			NLB
39	dup	1	p	10.3	Amniocentesis	NAD	Discordant-positive			Unconfirmed
40	dup	1	p(36.21-32.3)	37.7	Amniocentesis	NAD	Discordant-positive	NAD		Unconfirmed
41	dup	1	q(21.1-32.3)	70.3	Amniocentesis	NAD	Discordant-positive	NAD	Uterine fibroids	Unconfirmed
42	dup	1	p(13.2-44)	136.3	CVS	NAD	Discordant-positive			Unconfirmed

Supplementary Table 1. Details of cases of high-risk cfDNA results for segmental chromosomal imbalances.

Case	Type	Chromosome	Region	Size (Mb)	Diagnostic testing	Diagnostic Result	Classification	Parental Testing	Ultrasound anomalies	Outcome
43	dup	1	p(36.32 -34.1)	41	Amniocentesis	NAD	Discordant-positive	NAD		NLB
44	dup	1	p(35.1-32.3), p12-q32.1	18.6	Amniocentesis	NAD	Discordant-positive		Uterine fibroids	NLB
45	dup	2	q(14.3-37.2)	109.6	Declined		Unconfirmed	NAD		TOP
46	dup	3	q(26.1-26.31)	9.9	Amniocentesis	NAD	Discordant-positive	NAD		Unconfirmed
47	dup	3	p(24.3-22.3)	9.1	Amniocentesis	NAD	Discordant-positive		Uterine fibroid	NLB
48	dup	4	q(34.1-35.2	14.5	Amniocentesis	Dup 4q(34.1-35.2)	True-positive	NAD		TOP
49	dup	4	q(28.1-28.3)	8.3	Amniocentesis	NAD	Discordant-positive	NAD		NLB
50	dup	5	q	29.1	Amniocentesis	NAD	Discordant-positive			NLB
51	dup	5	p(14.3-13.3)	10	Amniocentesis	NAD	Discordant-positive	NAD		NLB
52	dup	5	p(15.33-14.1)	22.8	Amniocentesis	NAD	Discordant-positive	NAD		Unconfirmed
53	dup	5	q(21.3-31.1)	23.5	Amniocentesis	NAD	Discordant-positive			Unconfirmed
54	dup	5	p(14.3-13.3)	10	Amniocentesis	NAD	Discordant-positive			NLB
55	dup	7	p11.2-q36.3	97.7	Amniocentesis	CPM	Discordant-positive			NLB
56	dup	7	q(22.1-31.1)	11.1	Declined		Unconfirmed		Uterine fibroid	NLB
57	dup	8	q(13.2-24.3)	71.9	Amniocentesis	NAD	Discordant-positive	NAD		Unconfirmed
58	dup	8	q(11.1-24.3)	98.4	Amniocentesis	NAD	Discordant-positive	NAD		NLB
59	dup	8	p(23.1-12)	21.3	Amniocentesis	8Mb Del 8p(23.3-23.1), 21Mb Dup 8p(23.1-12)	True-positive	NAD	Cardiac anomalies; small RV, VSD	TOP
60	dup	8	p(23.1-11.22)	26.6	Amniocentesis	Inverted del-dup 8p syndrome	True-positive		Raised NT at 10/40, uterine fibroids	TOP
61	dup	11	q(11-25)	79.6	Amniocentesis	NAD	Discordant-positive	NAD		NLB
62	dup	11	q(23.2-24.1)	8.5	Amniocentesis	NAD	Discordant-positive	NAD		Unconfirmed
63	dup	13	q(14.2-34)	67	Declined		Unconfirmed			NLB
64	dup	13	q(21.31-21.33)	8.3	Declined		Unconfirmed	NAD	Uterine fibroids	NLB
65	dup	15	q(11.2-14)	13.7	Amniocentesis	NAD	Discordant-positive	NAD		Unconfirmed
66	dup	17	p(13.1-11.2)	8	Amniocentesis	NAD	Discordant-positive			NLB
67†	dup	18	q(11.2-23)		Amniocentesis	NAD	Discordant-positive	NAD		NLB
67†	dup	21	p11.2-q22.11		Amniocentesis	NAD	Discordant-positive	NAD		NLB

Supplementary Table 1. Details of cases of high-risk cfDNA results for segmental chromosomal imbalances.

Case	Type	Chromosome	Region	Size (Mb)	Diagnostic testing	Diagnostic Result	Classification	Parental Testing	Ultrasound anomalies	Outcome
68	dup	18	p(11.32-11.21)	15	CVS	1.6Mb Del Xp22.31, 15.0Mb Dup 18p(11.32-11.21)	True-positive		Crash sign, bilateral talipes, cystic lumbo-sacral structure	TOP
69	dup	18	p	10.9	Amniocentesis	NAD	Discordant-positive			Unconfirmed
70	dup	18	p(11.32-11.21)	14.4	Amniocentesis	NAD	Discordant-positive			Unconfirmed

†Women who received a high-risk result for multiple segmental imbalances involving different chromosomes

del=deletion, dup=duplication, NT=nuchal translucency, GA=gestational age, RV=right ventricle, VSD=ventricular septal defect, CVS=chorionic villus sampling, NAD=no anomaly detected, TOP=termination of pregnancy, NLB=normal live birth
